# Temporal Dimensions of Neighborhood Contexts and Cognitive Health

**DOI:** 10.1093/geroni/igaf122.543

**Published:** 2025-12-31

**Authors:** Elizabeth Muñoz

**Affiliations:** University of Texas at Austin, Austin, Texas, United States

## Abstract

Neighborhood environments are critical social determinants of cognitive health, yet they are structured in ways that advantage some groups while disadvantaging others. Investigating how neighborhoods shape cognitive function in racially and ethnically minoritized individuals is essential for informing policies and programs that address environmental inequities and promote cognitive health. While previous research has begun to demonstrate distinct associations between neighborhood domains— structural, physical, and social—and cognitive function, the temporal effects of neighborhood exposures are less clear. This presentation will explore the influence of neighborhood exposures across macro- and micro-time scales. At the macro level, I will summarize research demonstrating how neighborhood exposures across the life course—from childhood to adulthood—impact cognitive function, drawing on data from the Health and Retirement Study and the Colorado Adoption/Twin Study of Lifespan behavioral development and cognitive aging. At the micro level, I will demonstrate how neighborhood exposures shape cognitive performance in real time, focusing on the effects of neighborhood violence using ecological momentary assessment data from racially and ethnically diverse adults in the Effects of Stress on Cognitive Aging, Physiology, and Emotions study. I will conclude by discussing ongoing research that takes a within-group perspective on the daily lived experiences of Latino adults in Central Texas investigating how daily exposures within distinct neighborhoods shape cognitive performance in the moment and over time. By integrating macro- and micro-temporal perspectives, this presentation will highlight the complex and dynamic ways in which neighborhood contexts shape cognitive health, informing future research, prevention, and policy efforts.

